# Computational modeling of temporal and sequential dynamics of foraging decisions

**DOI:** 10.1186/1471-2202-15-S1-P137

**Published:** 2014-07-21

**Authors:** Kanghoon Jung, Hyeran Jang, Jerald D Kralik, Jaeseung Jeong

**Affiliations:** 1 Department of Bio and Brain Engineering, Korea Advanced Institute of Science and Technology (KAIST), Daejeon 305-701, Korea; 2 Department of Psychological and Brain Sciences, Dartmouth College, Hanover, NH 03755, USA

## Background

A fundamental understanding of behavior requires predicting when and what an individual will choose. However, the actual temporal and sequential dynamics of successive choices made among multiple alternatives remain unclear.

## Methods

In the current study, we tested the hypothesis that there is a general bursting property in both the timing and sequential patterns of foraging decisions. We conducted a foraging experiment in which rats chose among four different foods over a continuous two-week time period. Regarding when choices were made, we found bursts of rapidly occurring actions separated by time-varying inactive periods, partially based on a circadian rhythm.

## Results

Regarding what was chosen, we found sequential dynamics in affective choices characterized by two key features: (a) a highly biased choice distribution; and (b) preferential attachment, in which the animals were more likely to choose what they had previously chosen. To capture the temporal dynamics, we propose a dual-state model consisting of active and inactive states. We also introduce a satiation-attainment process for bursty activity, and a non-homogeneous Poisson process for longer inactivity between bursts. For the sequential dynamics, we propose a dual-control model consisting of goal-directed and habit systems, based on outcome valuation and choice history, respectively.

**Figure 1:**
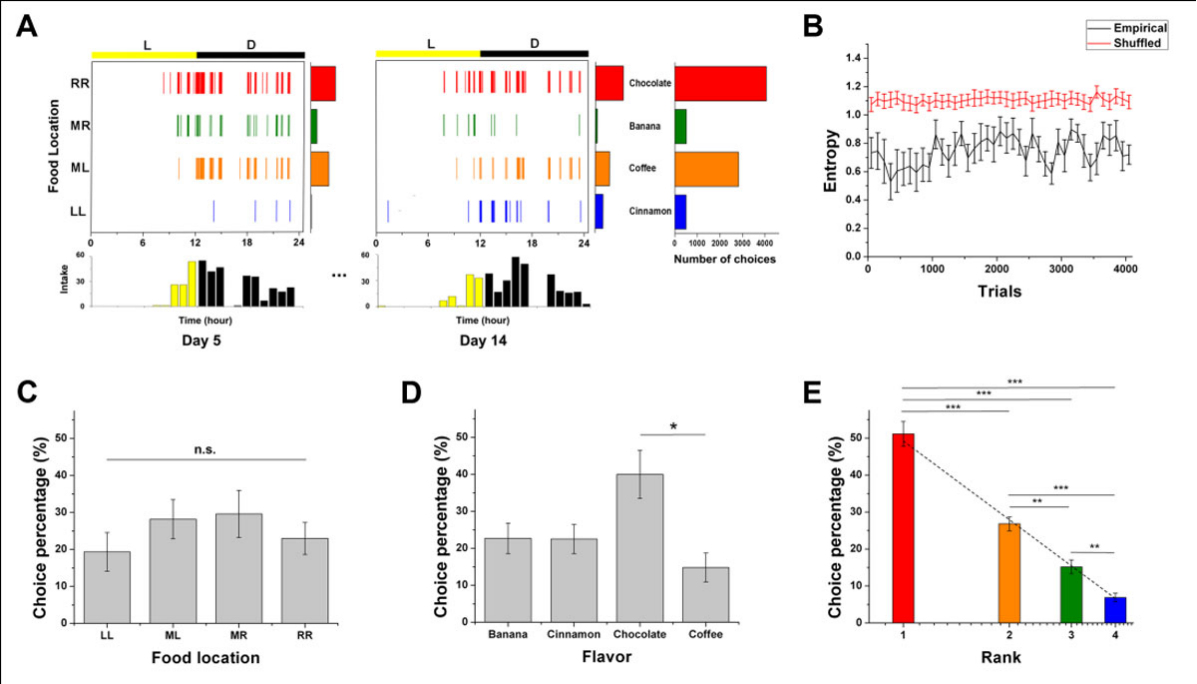
An example of the empirical choice patterns and the mean choice percentage of consumed pellets by food location, flavor, and rank.

**Figure 2:**
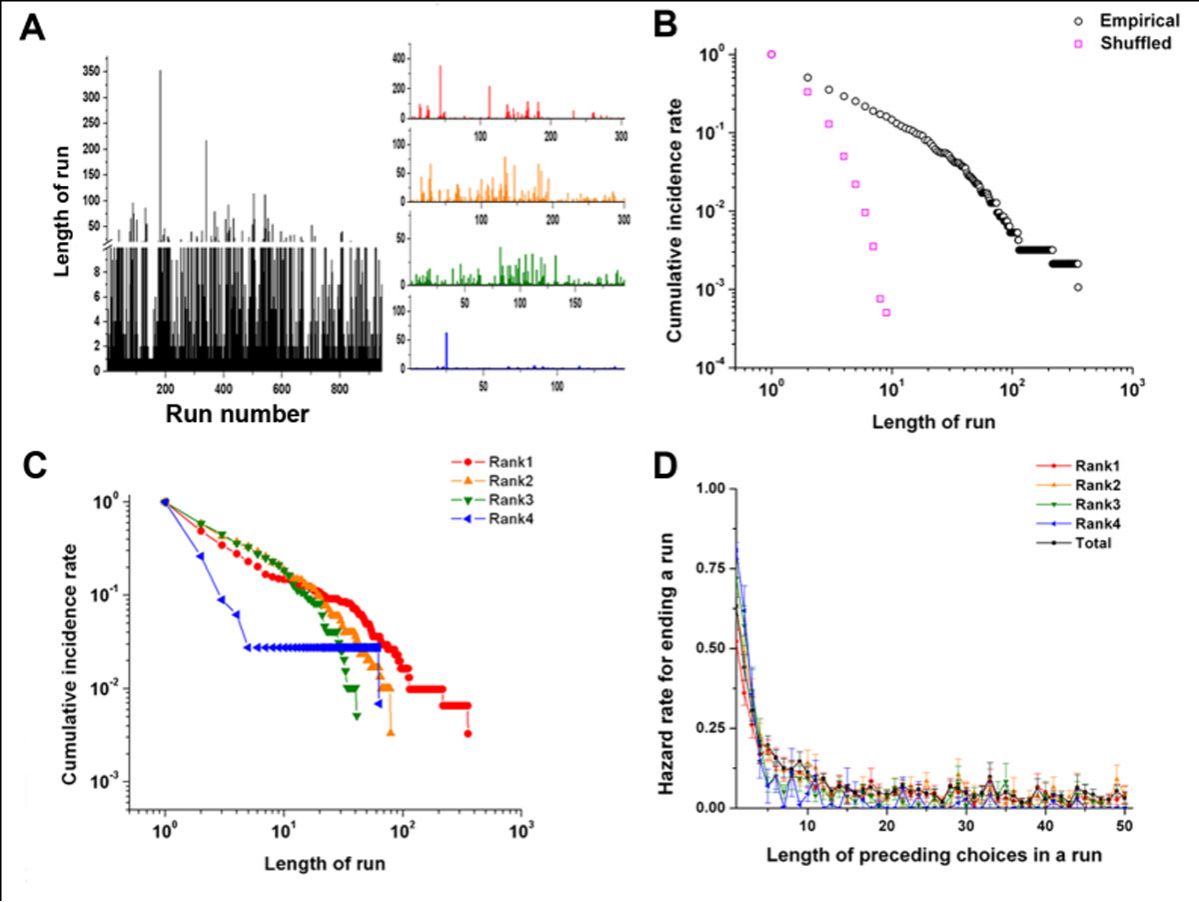
Temporal features of the foraging behavior.

**Figure 3:**
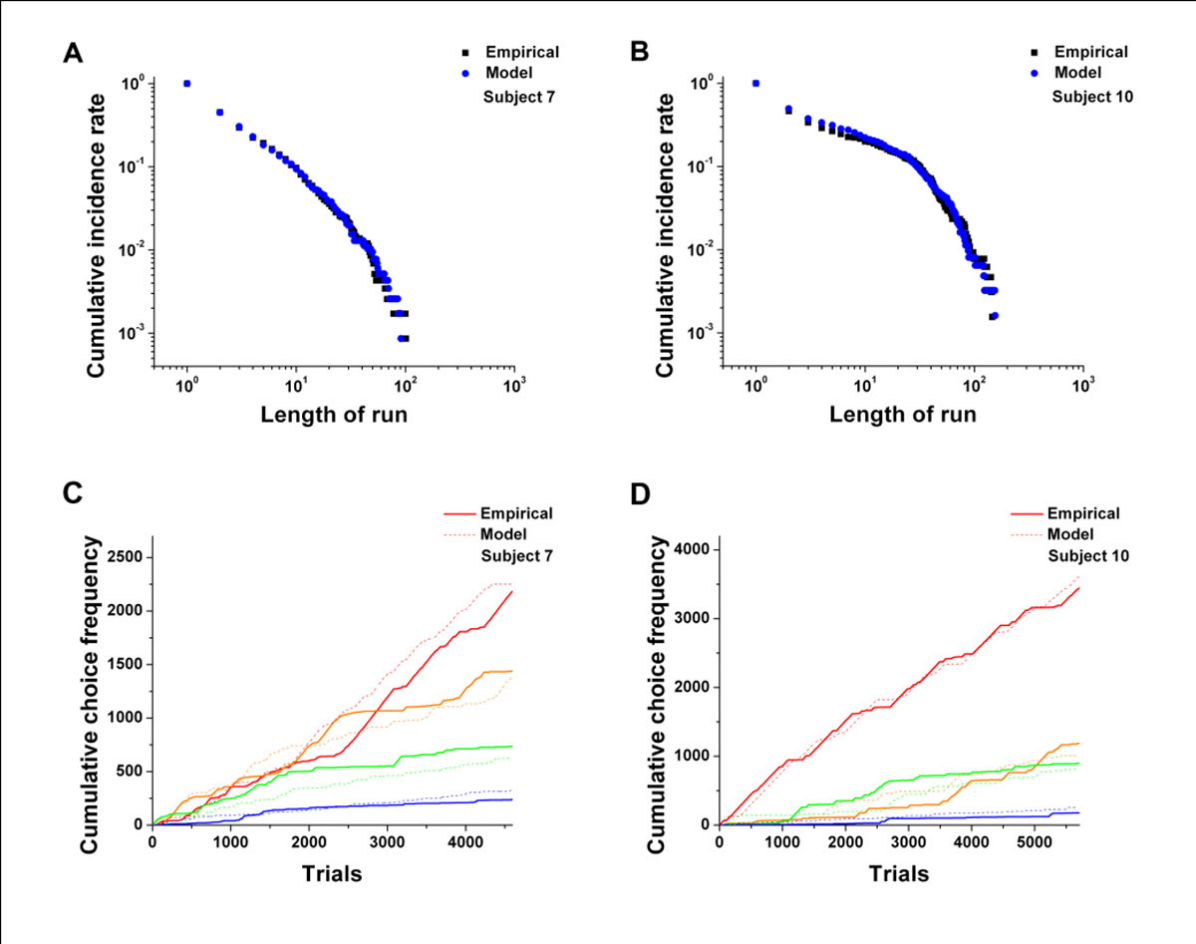
Comparison of a choice sequence generated from the dual-control model with the empirical data from two representative rats.

## Conclusions

This study provides insights into how the bursty nature of behavior emerges from the interaction of different underlying systems, leading to heavy tails in the distribution of behavior over time and choices.
